# Identification and transfer to stepped care of depressed and psychosocially stressed parents during peri- and postpartum—UPlusE: study protocol for cluster randomized trial of a screening intervention

**DOI:** 10.1186/s13063-024-08610-y

**Published:** 2024-11-14

**Authors:** Ulrike Stentzel, Neeltje van den Berg, Freya Lanczik, Andrea Gehrmann, Ina Nehring, Volker Mall, Anna Friedmann, Carolin Seivert, Stefanie Schade, Christoph Fusch, UPlusE-group, Neeltje van den Berg, Andrea Gehrmann, Volker Mall, Christoph Fusch, Ines Bauer, Anke Emgenbroich, Tilo Radau, Sebastian Jonas-Dieke, Sean Monks, Sarah Kittel-Schneider, Sarah Kittel-Schneider, Susanne Simen

**Affiliations:** 1 Institute for Community Medicine, University Medicine Greifswald, Ellernholzstr. 1-2, Greifswald, 17475 Germany; 2 German Center for Child and Adolescent Health (DZKJ), partner site Greifswald/Rostock, Greifswald, Germany; 3 Zentrum für Psychische Gesundheit (ZEP), Department of Psychiatry, Psychotherapy and Psychosomatics at the University Hospital Würzburg, Universitätsklinikum Würzburg, Würzburg, Germany; 4 Lehrstuhl für Sozialpädiatrie, Technische Universität, München, Chair of Social Paediatrics at the Technical University of Munich, Munich, Germany; 5 German Center for Child and Adolescent Health (DZKJ), partner site Munich, Munich, Germany; 6 Universitätsklinik für Psychiatrie und Psychotherapie Nuremberg, Nuremberg Clinic and Paracelsus Medical University (PMU), Nuremberg, Germany; 7 Universitätsklinik für Neugeborene, Kinder und Jugendliche Nuremberg, Nuremberg Clinic and Paracelsus Medical University (PMU), Nuremberg, Germany; 8 Department of Psychiatry and Neurobehavioural Science, University College Cork, Cork, Ireland; 9 APC Microbiome, University College Cork, Cork, Ireland

**Keywords:** Postnatal depression, Psychosocial stress, Screening, Mental health, Infants, Cost-effectiveness, Regulatory problems, EPDS, PBQ, Utilization

## Abstract

**Background:**

Perinatal depression affects 10–15% of mothers and approximately 5% of fathers. However, only a small number of affected individuals seek treatment. If left unrecognized and untreated, it can have negative long-term consequences for the family’s health, leading to subsequent high costs. Early treatment is crucial, yet there is a notable underdiagnosis and undertreatment. Affected individuals are often seen during this time, e.g. in paediatric practices, but not by specialists in mental health. Consequently, this study aims to increase detection and treatment rates of affected individuals by implementing a screening for depression and psychosocial stress in perinatal and postpartum parents within routine obstetric and paediatric care with subsequent advice and—if necessary—further referral to a mental health specialist.

**Methods:**

UPlusE is a prospective, cluster-randomized controlled trial conducted in an outpatient setting. Obstetric and paediatric practices will be randomized into an intervention and control group (1:1 ratio). Practices and enrolling patients will be required to use specific smartphone apps (practice apps) for interaction. The screening will occur with the apps at each paediatric checkup up to the child’s age of 12 months, using the Edinburgh Postnatal Depression Scale (EPDS), KID-PROTEKT questionnaire, and the scale 1 (impaired bonding) of the Postpartum Bonding Questionnaire (PBQ-1). The goal is to screen 10,000 patients across Germany. Gynaecologists and paediatricians will receive certified training on peripartum depression. Participants in the intervention group with scores above cut-offs (EPDS ≥ 10, KID-PROTEKT ≥ 1, PBQ-1 ≥ 12) will receive counselling through their treating gynaecologists/paediatricians and will be provided with regional addresses for psychiatrists, psychotherapists, and “Frühe Hilfen” (early prevention) as well as family counselling centres, depending on symptom severity. At each screening, participants will be asked whether they sought support, where, and with whom (utilization). Utilization is the primary outcome.

**Discussion:**

The screening is designed to reduce underdiagnosis to enable suitable support at an early stage (especially for those often overlooked, such as individuals with “high-functioning depression”) and hence to avoid manifestation of mental health problems in the whole family, especially infants who are exceptionally dependent on their parents and their well-being will benefit from this program.

**Trial registration:**

German Clinical Trials Register, DRKS00033385. Registered on 15 January 2024.

## Administrative information

Note: the numbers in curly brackets in this protocol refer to SPIRIT checklist item numbers. The order of the items has been modified to group similar items (see http://www.equator-network.org/reporting-guidelines/spirit-2013-statement-defining-standard-protocol-items-for-clinical-trials/).

**Table:** 

Title {1}	Identification and transfer to stepped care of depressed and psychosocially stressed parents during peri- and postpartum—UPlusE: study protocol for cluster randomized trial of a screening-intervention.
Trial registration {2a and 2b}.	German Clinical Trials Register (DRKS00033385) on 15^th^ January 2024
Protocol version {3}	Original first version, 15^th^ July 2024
Funding {4}	The study is funded by the Innovation Fund of the Federal Joint Committee (G-BA) in the funding program “New forms of care”. Funding ID: 01NVF22115. The funding period is set between 1^st^ August 2023 to 31^st^ December 2026.
Author details {5a}	Ulrike Stentze^l^ ulrike.stentzel@uni-greifswald.de (First and corresponding author) Neeltje van den Berg^1^ neeltje.vandenberg@uni-greifswald.de Freya Lanczik^2^ Lanczik_F@ukw.de Andrea Gehrmann^2^ Gehrmann_A@ukw.de Ina Nehring^4,5^ Ina.Nehring@tum.de Volker Mall^4,5^ volker.mall@tum.de Anna Friedmann^4,5^ anna.friedmann@tum.de Carolin Seivert^6^ Carolin.Seivert@klinikum-nuernberg.de Stefanie Schade^1^ stefanie.schade@med.uni-greifswald.de Christopgh.Fusch^7^ Christoph.fusch@klinikum-nuernberg.de Sarah Kittel-Schneider^2,8,9^ SKittelSchneider@ucc.ie Susanne Simen^6^ Susanne.Simen@klinikum-nuernberg.de ^1^Institute for Community Medicine, University Medicine Greifswald, Ellernholzstr. 1–2, 17,475 Greifswald. ^2^German Center for Child and Adolescent Health (DZKJ), partner site Greifswald/Rostock, Greifswald, Germany ^3^Zentrum für Psychische Gesundheit (ZEP), Universitätsklinikum Würzburg—Department of Psychiatry, Psychotherapy, and Psychosomatics at the University Hospital Würzburg ^4^Lehrstuhl für Sozialpädiatrie; Technische Universität, München, Chair of Social Paediatrics at the Technical University of Munich ^5^German Center for Child and Adolescent Health (DZKJ), partner site Munich ^6^Universitätsklinik für Psychiatrie und Psychotherapie Nuremberg, Nuremberg Clinic and Paracelsus Medical University (PMU) ^7^Universitätsklinik für Neugeborene, Kinder und Jugendliche Nuremberg, Nuremberg Clinic and Paracelsus Medical University (PMU) ^8^Department of Psychiatry and Neurobehavioural Science, University College Cork, Cork, Ireland. ^9^APC Microbiome, University College Cork, Cork, Ireland
Name and contact information for the trial sponsor {5b}	Department of Psychiatry and Psychotherapy, Nuremberg Clinic, Paracelsus Medical University Klinikum Nürnberg Süd, Breslauer Straße 201, 90,471 Nürnberg, Germany Dr. Susanne Simen (Susanne.Simen@klinikum-nuernberg.de)
Role of sponsor {5c}	The study sponsor initiated the study and is responsible for coordinating it. The funding body was not actively involved in the study design or will be in the data collection or analysis or interpretation or publication of the results.

## Introduction

### Background and rationale {6a}

A good start in life can be significantly compromised by a parent’s mental illness. Perinatal depression has a prevalence of 10–15% among mothers [1] and approximately 5% among fathers [2]. Psychosocial stressors (including depression) are reported by 30% of parents [3]. The prevalence of suicidal thoughts during pregnancy and postpartum ranges from 5 to 14% [4]. Suicide is one of the leading causes of death among mothers in the first year after childbirth [4]. However, significantly fewer individuals affected by a mental illness (like for example depression and anxiety disorders as the most common conditions) seek treatment during the perinatal period compared to other life phases [5]. Left unrecognized and untreated, both conditions can have negative long-term consequences for the family’s health and lead to high subsequent costs [6–8]. These illnesses often become chronic [9], resulting in impairments in sensitive caregiving for the child, attachment disorders, child regulatory problems, and, in the worst case, emotional neglect and abuse [10–12]. Early treatment of depression reduces individual suffering [1], enhances parenting skills [13], and reduces healthcare system costs [14–17]. Nevertheless, only a fraction of affected patients are diagnosed and treated early; due to lack of awareness and stigma, there is still a pronounced underdiagnosis and undertreatment [5, 18]. Gynaecologists and paediatricians regularly encounter individuals in this phase of life but frequently fail to recognize their psychological burdens [18]. For instance, there are highly functional affected individuals who appear capable and healthy, even though they are severely ill. Without a screening in routine care in these cases, mental illnesses often go unnoticed—with all the aforementioned risks. To reliably and promptly identify mothers and fathers with perinatal mental illnesses screening by gynaecologists and paediatricians is meaningful, feasible, and urgently needed [4, 14, 19–21]. So far, there are no general, systematic, and standardized early detection programs in Germany, and timely, low-threshold, and tailored provision of support is not organized. While the current guidelines for children and maternity care of the German Federal Joint Committee* (Kinder- und Mutterschafts-Richtlinien des* Gemeinsamen Bundesausschusses (G-BA)) partially consider the need to recognize psychosocial burdens, these risk factors are only identified in individual cases, as binding and feasible standards for required assessment are lacking so far. Hence, the identification of mental illnesses, such as depression, in pregnant women and parents is not yet a part of routine care. This aspect will be addressed by the G-BA-funded project “UPlusE (U-Untersuchung für Kinder PLUS Eltern beim Pädiater zur Förderung der kindlichen Entwicklung mit Impuls aus frauenärztlicher Schwangerenvorsorge”—Paediatric Check-up for Children PLUS Parents at the Paediatrician to Promote Child Development with Input from Gynaecological Prenatal Care). Within UPlusE, a systematic and very low-threshold screening for peripartum/postnatal depression or high psychosocial stress in (A) pregnant women during the third trimester by their treating gynaecologists and in (B) parents during paediatric checkups (so called U-checkups) for their children (up to U6-checkup at age of 10–12 months) by their treating paediatricians will be conducted throughout Germany. In the case of a positive screening result, the affected parents will be referred by their respective doctors for appropriate support (including psychiatry, psychotherapy, and psychosomatics treatments, hereafter referred to as “mental health specialists”) and/or to “early prevention” and family support programs (including early prevention services *Frühe Hilfen*, *pro familia*, and family and parenting counselling centres, pregnancy counselling centre, child protection coordination centres as well peer support groups).

## Objectives {7}

The overall goal of UPlusE is to increase the utilization of treatment and counselling services for mothers and fathers experiencing perinatal depression, psychosocial distress, or impaired bonding preceded by a low-threshold screening for these conditions. Within UPlusE, the term psychosocial stress encompasses both psychosocial stress and impaired bonding. The main research question of the UPlusE project is: can the utilization of any support services by parents with psychological or psychosocial issues be improved through the low-threshold intervention? The working hypothesis is: there is a significantly higher utilization of support services in the intervention group between enrolment in the project and the U6 checkup (child’s age of 10–12 months) compared to the control group.

Additional secondary research questions:

1. For parents positively screened for peripartum depression or psychosocial distress (in the following meant as psychosocial distress and impaired bonding):
  a What mental health services are utilized? What is the proportion of medication-based therapies? How quickly do they receive treatment?
  b How frequently do various forms of child regulatory problems occur in this group?
  c Do child regulatory problems occur with varying frequency in families with different stress profiles (mental illness/psychosocial burdens)?

Sensitivity analyses will investigate the following questions:

iv. Is the proportion of parents experiencing an improvement in depressive symptom severity higher when utilizing mental health services?
v. Is the proportion of parents with an unaffected parent–child relationship higher when utilizing mental health services?
vi. Do child regulatory problems occur less frequently in parents utilizing mental health services?

2. Comparison between intervention and control groups at the time of U6 (child’s age of 10–12 months) to answer the following questions:
  a Is the proportion of parents without depressive symptoms greater in the intervention group?
  b Is the proportion of parents with an improvement in depressive symptom severity higher in the intervention group?
  c Is the proportion of parents with an unaffected parent-child relationship higher in the intervention group?
  d Is the proportion of parents with an improvement in parent-child relationship higher in the intervention group?
  e Is the proportion of parents with psychosocial burdens smaller in the intervention group?
  f Is the proportion of children with regulatory problems in areas of crying, sleeping, and feeding smaller in the intervention group?
3. For all groups (differentiated by positively and negatively screened parents):
  a Depressive symptoms, psychosocial burden, parent–child relationship, and child regulation: what is the trajectory of impairment values in different paediatric examinations?
  b Do child regulatory problems occur more frequently in children of positively screened parents than in children of negatively screened parents?
  c Do parents feel stigmatized by the screening or the intervention?
  d What healthcare services are utilized in addition to mental health services?
  e What are the healthcare system costs in the first year after birth?
  f What is the acceptance among participating parents for screening and intervention?
  g How long is the waiting time until appointments for support services?

Questions at the level of participating practices:

h. How well is the screening and intervention (counselling parents with mental health/psychosocial issues and encouraging them to utilize the services of mental health practitioners or support services) accepted among paediatricians and gynaecologists? Where are the potential barriers?
i. Do paediatricians and gynaecologists perceive the offered training as helpful and meaningful?

Additionally, as secondary outcomes experiences of violence and birth experiences (fulfilling–traumatic) will be evaluated.

## Trial design {8}

**Fig. 1:**
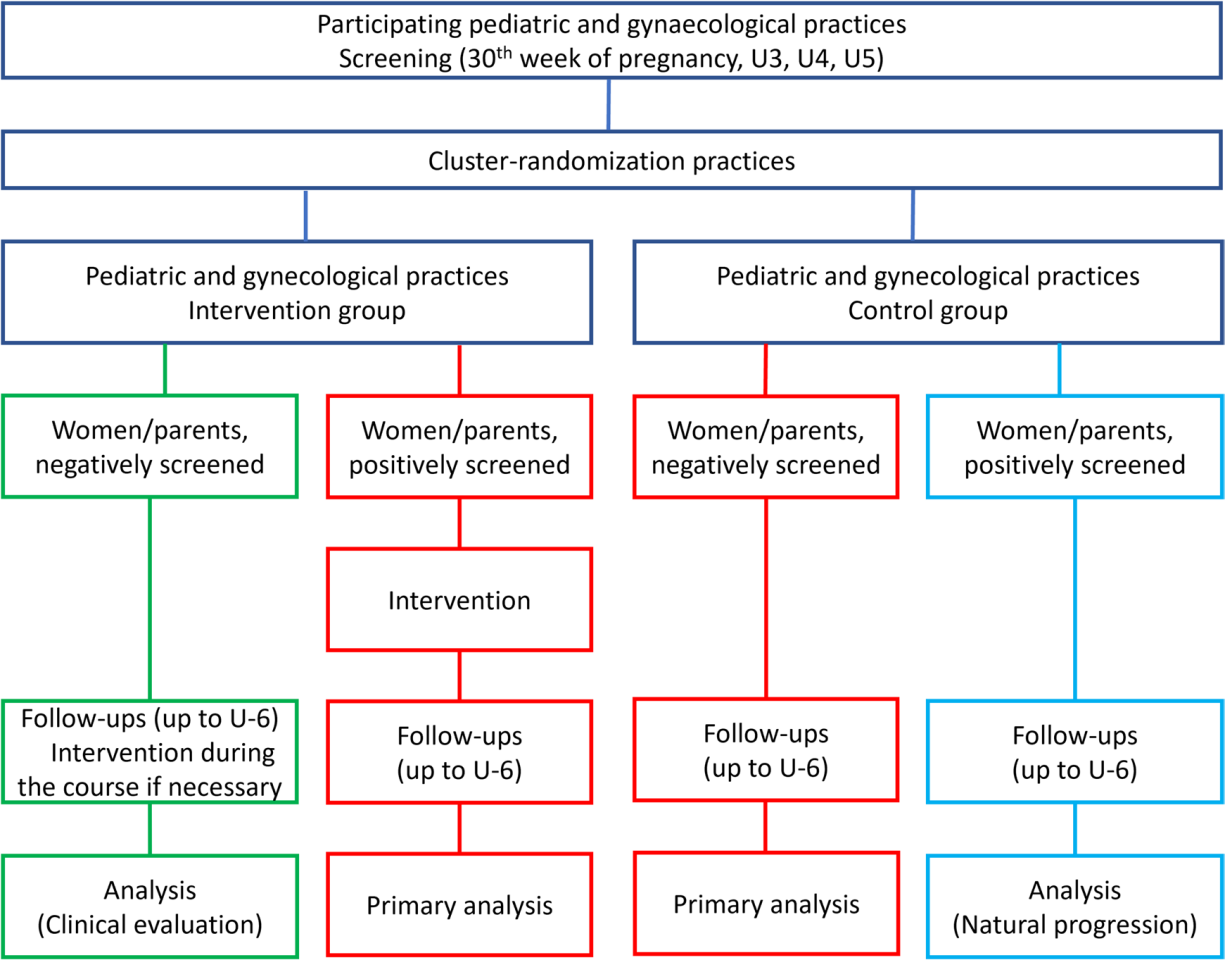
UPlusE design

UPlusE is a prospective, cluster-randomized controlled trial conducted in an outpatient setting. The intervention (screening and, if indicated, consultation by treating doctors) is a new form of healthcare provision. In line with this, UPlusE is being implemented and evaluated within the normal healthcare context. UPlusE is an experimental study within a superiority framework. The effects of the UPlusE intervention will be evaluated by comparing the intervention group with the control group, which receives treatment as usual. If the intervention works as intended, the intervention group will have better values for primary and secondary outcomes. The practices are randomized into an intervention and a control group (1:1 ratio, ensuring an equal distribution of 50% to both groups). The cluster randomization is chosen because the intervention will affect the organizational structure of the entire practice: this includes participant-specific, organizational, and implementation aspects. Thus, counselling parents with mental health/psychosocial issues and encouraging them to utilize the services of mental health practitioners or support services is a responsibility of the entire practice. The cluster randomization ensures that participants in each practice either receive the intervention or serve as the control group, depending on the practice’s assignment. Participants will be recruited nationally from participating paediatric and gynaecological practices. Through screening for depression and psychosocial stress, both groups are expected to yield positive (screened positive) and negative (screened negative) results. In the intervention group, participants receive the intervention as soon as a positive result occurs. Participants in the control group receive treatment as usual. For assessing the success of the intervention (clinical evaluation), comparing participants from both groups with positive results is relevant. Participants in the control group with negative results will additionally be observed concerning the natural course of events from the inclusion point up to U6. The study design is depicted in Fig. 1.

## Methods: participants, interventions, and outcomes

### Study setting {9}

UPlusE is a new module of an already established healthcare program (BKK Starke Kids) which offers health and preventive services for children and adolescents beyond regular healthcare to their customers. The UPlusE-module addresses the parents’ mental health and psycho-social distress. UPlusE will also be open to insured individuals from other insurers.

Outpatient gynaecologists and paediatricians throughout Germany will be recruited to participate in UPlusE with their practices. One requirement for the physicians to participate in UPlusE is the utilization of the practice apps “My Gynaecology Practice” (German: "Meine GynPraxis”) or “My Paediatric Practice” (German: "Meine pädiatrische Praxis”) in their practices. The apps are well-established communication tools connecting patients with their gynaecologists respectively parents with their paediatrists. Participating physicians will receive a certified Continuing Medical Education (CME) online training free of charge, focusing on managing the screening process and the management of peripartum mental health disorders.

### Eligibility criteria {10}

#### Inclusion criteria

All pregnant individuals from the 30th week of pregnancy onwards and parents (mothers OR fathers) of children up to 6 months of age (latest entry into the study at the U5-checkup (at child’s age 6 to 7 months) can participate in UPlusE.

Physicians can participate if they are gynaecologists or paediatricians using the practice app. Interventions by gynaecologists or paediatricians will include counselling and motivation of positive screened parents to seek help at support or mental health services. To ensure mental health treatment, a network of mental health specialists is founded, who gave their consent to take part in UPlusE and who will therefore be listed in the app.

#### Exclusion criteria

Exclusion criteria include lack of capacity to give consent or an inability to complete the questionnaires in the app (no smartphone, insufficient language, or reading skills). This study thus excludes individuals who cannot operate a smartphone and those who do not speak sufficient German. Many people who do not yet have a sufficient command of the German language use translation programs on their smartphones. If pregnant individuals/parents understand the informed consent and questionnaires adequately with the help of translation programs, they can naturally be included.

### Who will take informed consent? {26a}

UPlusE participants are required to install one of the aforementioned apps on their smartphones in order to take part. Pregnant individuals will be recruited and enrolled by participating gynaecologists, and parents will be recruited and enrolled by paediatricians. Enrolment is achieved through a digital signing of the consent form and data protection declaration within the practice apps. A comprehensive patient information document precedes the consent form. The consent forms are then digitally transmitted to the project management for documentation and compliance with retention obligations.

### Additional consent provisions for collection and use of participant data and biological specimens {26b}

In UPlusE, data is collected through questionnaires. Additionally, health insurance data from the participating health insurance company (58 BKKs) will be used to descriptively analyse the utilization of health system services and their costs. Detailed information about these data collection processes is provided in the patient information. The informed consent form, in which participants agree to participate, refers to this data collection. No other data is collected, and no biological samples or similar materials are taken.

## Interventions

### Explanation for the choice of comparators {6b}

Up to the final data collection point at U6, participants in the control group will receive regular healthcare (treatment as usual). This group will serve as the comparator for the intervention. At the conclusion of the study, they will be provided with the benefits of the intervention (feedback to the screening results and, if necessary, recommendations for further treatment).

### Intervention description {11a}

The intervention is delivered to participants through the practice apps. These apps are established communication tools used for conveying practice information and communication, scheduling, or reminding appointments.

With the UPlusE module, the following interventions are being conducted:

- All participants receive information/guidance about childbirth and parent–child interaction through automatically sent push notifications. The intervention group gets more specific guidance; the control group gets unspecific information.
- All participants are screened regularly for depression, psychosocial stress, and impaired bonding (in parents) through questionnaires.
- All participants in the intervention group receive their screening result, subsequent information about peripartum depression, psychosocial distress, distress with their babies, and recommendations for treatment and/or counselling on special pages of the website www.upluse.de.
- Participants in the intervention group with a positive screening result receive their screening result with subsequent recommendations for treatment and/or counselling from their treating physician. Simultaneously, regional contact information for mental health practitioners, “Early Prevention” and counselling services, peers, and support groups are made available in the apps’ data pool.

**Fig. 2:**
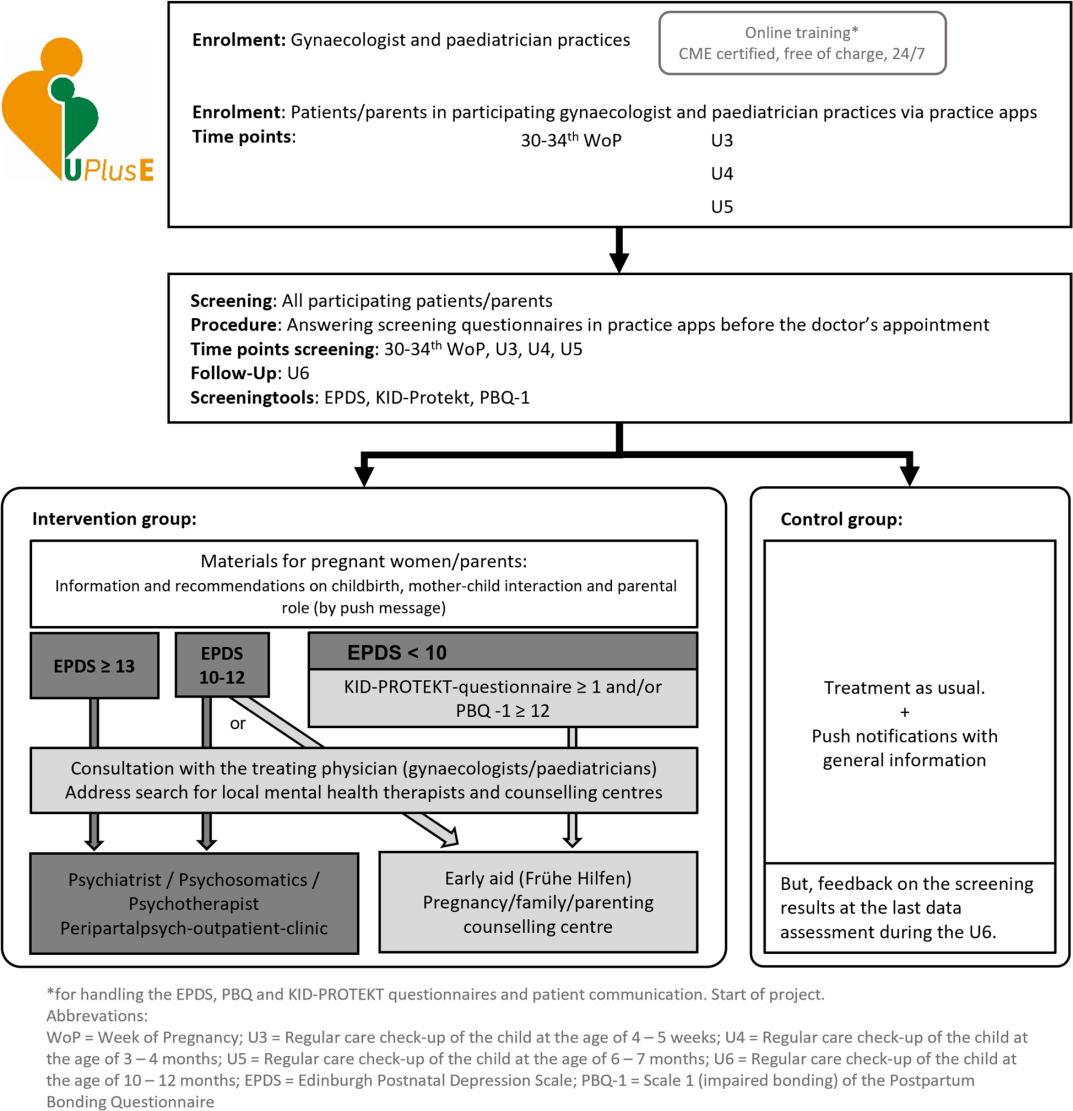
UPlusE intervention flow

Participants from practices in the intervention group will receive the result of the screening and suitable recommendations for support whenever positively screened during the observation period: pregnant individuals enrolled in gynaecologic practices or parents enrolled in paediatric practices who screen positively during their initial contact with UPlusE will promptly receive the recommendations. Pregnant individuals/parents in the intervention group who are initially screened as negative but are subsequently screened as positive during the course will receive the recommendations from that point onward. As screening tools, the Edinburgh Postnatal Depression Scale (EPDS), the KID-PROTEKT questionnaire, and the scale 1 (impaired bonding) of the Postpartum Bonding Questionnaire (PBQ) will serve. The intervention flow is depicted in Fig. 2. For an EPDS score ≥ 13, trained physicians should encourage the affected parents to promptly consult a mental health practitioner in the region as provided through the app. In case of an EPDS score of 10–12, it is recommended that individuals consider seeking assistance from mental health professionals or, alternatively, ‘Early Prevention’ services. In case of a PBQ scale 1, score ≥ 12, and/or KID-PROTEKT questionnaire ≥ 1 and EPDS < 10, physicians encourage the affected individuals to seek support from ‘Early Prevention’ and other counselling services. The relevant regional addresses will be provided to participants of the intervention group through the apps and the UPlusE website (www.upluse.de). If needed, the physician will support them in making contact. Before each U-checkup, questionnaires are sent again via the app, and the utilization of support services is assessed. The app itself does not guide physicians in treatment recommendations but rather replaces paper questionnaires and calculators to make the screening process more efficient and sustainable.

Parents in the control group (CG) receive the same questionnaires; however, patients of the CG get their results only at the U6 checkup at the child’s age of 10–12 months (end of the observation period), with regular care provided until then.

### Criteria for discontinuing or modifying allocated interventions {11b}

The intervention does not involve invasive or burdensome procedures. The control group receives usual care. Here, neither the doctor nor the patients receive the screening results before U6. If clinical indications of a mental disorder, psychosocial stress, or impaired bonding are identified during clinical assessments or at the final evaluation at U6, the individuals are also encouraged to seek support. In the control group, the same questionnaires are used at all measurement points like in the intervention group to obtain differentiated evaluation results.

If the question about suicidality is answered positively in the EPDS (the thought of harming myself has occurred to me: yes, quite often, or sometimes), the physician receives a note and will give a treatment recommendation immediately—regardless of control—or intervention group. The same procedure applies, when a specific question indicates a risk to the child’s well-being: the treating physician can immediately respond, initiate a conversation with the parents, and take appropriate measures. In general, both the control and intervention groups are shown general information in the app about whom to contact during acute crises and suicidal thoughts, regardless of questionnaire results.

The risk of stigma due to this screening appears to be very low. On the contrary, the training of doctors, systematic screening of all parents, and the information about the apps are intended to counteract stigma. The KID-PROTEKT screening questionnaire was developed in collaboration with affected individuals, and the wording was chosen to avoid stigmatization. Individuals retain their autonomy at all times regarding whether data is shared within the network and whether they accept assistance or seek psychological treatments.

The UPlusE intervention is purely advisory in nature and does not involve invasive elements. Therefore, the formulation of termination criteria or criteria for discontinuing or modifying allocated interventions is not necessary. Nevertheless, every patient who wishes to quit the study can do so at any time.

### Strategies to improve adherence to interventions {11c}

UPlusE is a module of a special care program (BKK Starke Kids) offered by the participating health insurance companies (58 BKKs). When patients in Germany choose to participate in a special care program, they receive clear advantages in their healthcare but also enter into legally binding obligations. With UPlusE, the obligation entails committing to their treating doctor for the duration of the care and filling out the questionnaires in the practice apps. Since the UPlusE study evaluates the UPlusE healthcare provision form, the study and the healthcare provision program are inseparable. Therefore, the legal regulations for the special care program apply here at least for BKK-insured individuals. Participants who no longer wish to take part in the study after more than 2 weeks must terminate their participation in the special care program. Until then, the obligation to complete the questionnaires remains. When it is time to fill out the questionnaires, participants will receive a push notification prompting them to do so. In case the questionnaire completion is forgotten or omitted, a reminder will be triggered.

### Relevant concomitant care permitted or prohibited during the trial {11d}

All participants will receive treatment as usual in addition to the study intervention. There are no treatments or interventions that would be prohibited during the study.

### Provisions for post-trial care {30}

UPlusE is a program for healthcare provision that is being investigated within the real healthcare setting with the aim of integrating it into regular care. Damages due to the intervention are not anticipated due to the non-invasive nature of the intervention. Therefore, we expect that compensation for any incurred damages will not be necessary.

### Outcomes {12}

The primary and secondary endpoints, as well as the parameters of the process evaluation, are presented in Table 1 along with the time points for assessment and measurement methods.

**Table 1:** Primary and secondary outcomes UPlusE evaluation

Primary endpoint	Measurement	Time points	Methods
Utilization of services from mental health practitioners/"Early Prevention"	Project-specific questionnaire	From inclusion to U6 checkup	Apps ‘My Paediatric Practice’, ‘My Gynaecology Practice’, all participating parents
Secondary endpoints			
Depression/severity of depression	EPDS (Edinburgh Postnatal Depression Scale)	From inclusion to U6 checkup	Apps (as above), all participating parents
Parent–child bonding	PBQ (Postpartum Bonding Questionnaire)	From inclusion to U6 checkup (child's age of 10–12 months)	Apps (as above), all participating parents
Psychosocial burden	KID-PROTEKT questionnaire	From inclusion to U6 checkup (child’s age of 10–12 months)	Apps (as above), all participating parents
Child regulation	Extended SFS questionnaire	From inclusion to U6 checkup (child’s age of 10–12 months)	Apps (as above), all participating parents
Stigmatization	Project-specific questionnaire	Inclusion and U6 checkup (child’s age of 10–12 months)	Apps (as above), all participating parents
Process evaluation, participant acceptance/satisfaction	Project-specific questionnaire	At U6 checkup (child’s age of 10–12 months)	Apps (as above), parents in the intervention group
Process evaluation, acceptance/satisfaction of participating practices	Project-specific questionnaire	After completion of implementation phase (months 33–35)	Apps (as above), all participating gynaecological and paediatric practices
Process evaluation, acceptance/satisfaction of mental health practitioners/ ‘Early Prevention’	Project-specific questionnaire	After completion of implementation phase (months 33–35)	All involved mental health practitioners/ ‘Early Prevention’ services
Utilization of services in the healthcare system	Analysis of data from the participating health insurance funds	After completion of implementation phase	Data from all participating parents and children
Healthcare system costs in the first year after childbirth	Analysis of data from the participating health insurance funds	After completion of implementation phase	Data from all participating parents and children

All questionnaires for all data assessments time points will be made available in the apps ‘My Gynaecology Practice’ and ‘My Paediatric Practice’, and parents will be prompted to fill them out a few days before each U-checkup appointment.

### Participant timeline {13}

**Fig. 3:**
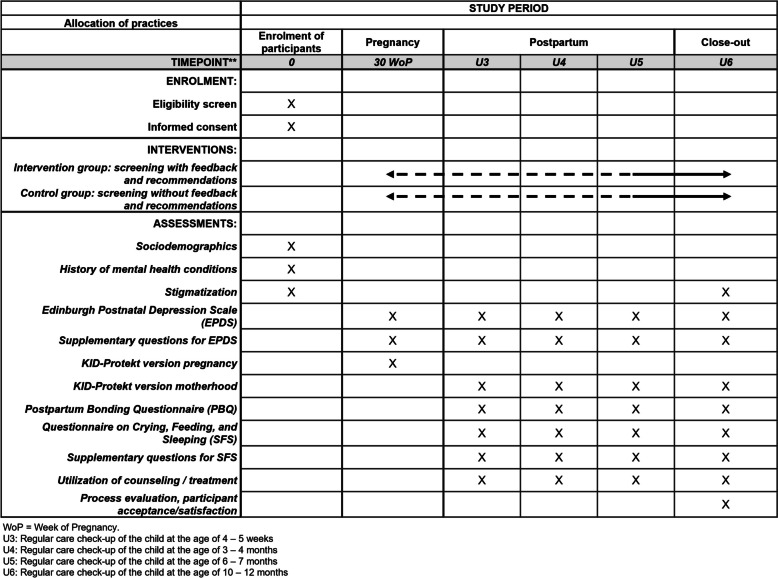
UPlusE schedule of enrolment, interventions, and assessments (SPIRIT)

Depending on entry point (enrolment into the study during pregnancy or at U3-, U4- or U5-checkup) and potential screening point at which a participant in the intervention group screens positive and is motivated for treatment, the duration of the intervention with follow-up observation varies between 6 and 15 months. The intervention is carried out up the U6-checkup (at child’s age 10 to 12 months). The participant timeline is depicted in Fig. 3.

### Sample size {14}

The proportion of all parents who avail themselves of any mental health services is based on the available figures. (18% [22], health insurance data: 10–12%) and is assumed to be 15% in the following. The assumption is that through the intervention, the proportion of affected parents using mental health services can be increased to at least 25% in the intervention group. With *α* < 0.05 and a power of 0.80, a sample size of 500 subjects is therefore required. The cluster-randomized controlled design must be considered when calculating the required number of participants. The design effect due to cluster randomization is estimated at 1.725 (intra-cluster correlation (ICC): 0.15; number of patients/practices: 2.5). With a dropout rate of 20%, the number of required positively screened subjects is (500 × 1.725) / 0.80 = 1078. Given a known prevalence of peripartum depression of 10–15% as the condition that is in our focus, a total of 10,000 parents (5000 each in the intervention and control groups) must be screened and included (Fig. 3).

### Recruitment {15}

With the current usage of the ‘My Paediatric Practice’ app by 750,000 parents and a daily increase of approximately 1000 parents, we consider achieving the required sample size as very realistic. UPlusE is a module of the BKK health care program ‘Strong Kids’ but will still be open to insured individuals from other insurers.

## Assignment of interventions: allocation

### Sequence generation {16a}

Following the confirmation of physicians’ participation in UPlusE, practices are randomly assigned to either the intervention or the control group (cluster randomization). The confirmation and randomization processes take place automatically within the practice app administration (opt-out regulation), with separate procedures for gynaecologists and paediatricians. The allocation to the groups is maintained at a 1:1 ratio, ensuring an equal distribution of 50% of physicians to both the intervention and control groups. To achieve this, randomization lists are generated in advance by the evaluation team using statistical software SAS 9.4 (TS Level 1M7 Copyright © 2016 by SAS Institute Inc., Cary, NC, USA) employing the method of simple random sampling (proc surveyselect). Participating practices are included in these randomization lists. To maintain a balanced ratio of intervention to control practices within regions, the inclusion in the list is organized by postal code. The automated nature of the randomization process precludes any external influence on its outcome.

### Concealment mechanism {16b}

The automated nature of the randomization process precludes any external influence on its outcome. Hence, there is no possibility to manipulate the allocation.

### Implementation {16c}

Each participant in a given practice will be designated to either receive the intervention or serve as part of the control group based on the practice’s assignment.

## Assignment of interventions: blinding

### Who will be blinded {17a}

Due to the interventional nature and the cluster-randomized design, blinding of nurses, physicians, and participants is not possible. They will become aware of their randomization status. Hence, this is an open, randomized study.

### Procedure for unblinding if needed {17b}

A procedure for unblinding is not applicable due to the open design.

## Data collection and management

### Plans for assessment and collection of outcomes {18a}

The following questionnaires will be used:

1. Socio-demographic questionnaire:
  Upon study enrolment, comprehensive socio-demographic data will be collected, including age, gender, number of pregnancies and births, history of previous mental health issues, partnership, education, occupation, and financial situation.
2. Edinburgh Postnatal Depression Scale (EPDS):
  The Edinburgh Postnatal Depression Scale (EPDS), an internationally validated screening instrument for depression [23], will be used. The scale consists of ten self-reported items about depressive symptoms. An EPDS total score of ≥ 10 points indicates a moderate probability of depression, while a score of ≥ 13 points indicates the presence of depression with high sensitivity and specificity [24], also suggesting the presence of anxiety disorders. In UPlusE, an adapted and pilot-tested version of EPDS (EPDS+) [25] will be employed.

The adapted version contains three more questions (‘Plus questions’):

- Have you ever experienced violence? Yes, quite often. Sometimes. Barely. Never.
- The birth of my child I experienced as…: Fulfilling. Okay. Unexpectedly difficult/with complications. Traumatizing.
- I have experienced my pregnancy (so far) as: Burdensome/tense. Rather burdensome/tense. Rather positive/relaxed. Positive/relaxed.

The ‘Plus questions’ do not influence the EPDS score, rather they can be interpreted as a short screening for traumatic experiences in addition to the screening for depressive symptoms [25].

3. KID-PROTEKT:
  The KID-PROTEKT questionnaire [3] will be used as a measuring instrument for parental psychosocial burdens. The questionnaire includes six items about personal stress situations. A score ≥ 1 is considered a basis for conversation to determine if there is a need for support .
4. Postpartum Bonding Questionnaire (PBQ):
  Additionally, the scale 1 (impaired bonding) of the Postpartum Bonding Questionnaire (PBQ) with 12 items will be used. Overall, the PBQ is a simple and well-validated instrument for assessing impaired parent-to-child bonding. The scale 1 is a general factor that signals the likelihood of a relationship problem at a threshold (cutoff value) of 12 points [26]. Responses to each of the 12 items will be given on a 6-point Likert scale (‘always’, ‘very often’, ‘quite often’, ‘sometimes’, ‘rarely’, and ‘never’) [27].
5. Questionnaire on Crying, Feeding, and Sleeping (SFS):
  For measuring infant regulatory disorders, the extended SFS questionnaire will be used, comprising 37 items about infant crying, sleeping, and eating behaviour [28]. Total mean scores above 1.84 (crying and sleeping) or above 1.27 (feeding scale) indicate regulatory problems. Six items based on DC: 0–5 (classification system for the diagnosis of mental disorders in infants, toddlers, and preschool children) will be used for further differentiation and clinical classification of symptoms related to excessive crying, sleep disorders, and early childhood feeding disorders.
6. Questionnaire on treatment/counselling utilization:
  Utilization of treatment for mental health issues and counselling for psycho-social stress and parent-child bonding issues will be systematically captured via a project-specific questionnaire in the practice apps ‘My Gynaecology Practice’ and ‘My Paediatric Practice’ through self-report by the participants. The questionnaire assesses information about whether treatment/counselling has been sought. If utilization has occurred since the previous assessment, details such as the institution visited (pregnancy counselling centre, child protection coordination centres, ‘Early Prevention’ services, specialized outpatient clinic, psychiatrist/psychosomatics/psychotherapist, general practitioner, other), the motivating factor, and the referrer will be collected. As of our knowledge, specifically fitting standardized, validated, and publicly published questionnaires for this purpose are currently not available.
7. Process analysis questionnaire
  To identify technical and organizational barriers as well as facilitating factors for the implementation of the care model, a process evaluation will be conducted. This involves surveying all participating practices, mental health practitioners, and counselling services. Except for mental health practitioners and ‘Early Prevention’, the survey will be conducted using project-specific questionnaires via the practice apps ‘My Gynaecology Practice’ or ‘My Paediatric Practice’. Mental health practitioners and ‘Early Prevention’ services cannot be reached through the practice apps: for the process evaluation, these recipients will be invited to participate in the survey via email by the responsible contact persons from the study team. The survey will use a digital form. The questionnaires for process evaluation will be designed by the Institute for Community Medicine, University Medicine Greifswald, and will cover aspects such as:
  The number of participating practices, mental health practitioners, and ‘Early Prevention’ services.
  Their reasons for participation.
  The feasibility of implementing the intervention in their respective settings.
  Acceptance of the intervention.
  Perceived technical and/or organizational barriers or gaps in the implementation of the intervention.
  Benefits perceived by the involved practices, mental health practitioners, and ‘Early Prevention’ services due to the intervention,
  Factors promoting the implementation of the care model.

The various aspects will likely be queried using five-point Likert scales ranging from ‘Strongly disagree’ to ‘Strongly agree’ or ‘Not at all satisfied’ to ‘Completely satisfied.’

### Plans to promote participant retention and complete follow-up {18b}

UPlusE is a module of a special health insurance care program (BKK Starke Kids) not only offering clear advantages in healthcare but also leading to legally binding obligations at least for BKK-insured individuals. With UPlusE, the obligation entails committing to their treating doctor for the duration of the care and filling out the questionnaires in the practice apps. The number of questionnaires per time will stay within reasons and will approximately take 10–15 min. Participants can submit their answers only after all questions have been answered. To further remind and support the participants, a weekly note is sent via push message, e.g. how can I talk to my baby, etc. UPlusE will also be open to insured individuals from other insurers.

### Data management {19}

The data collected from the questionnaires through the app are managed in a secure and protected data management system. The telemedical service provider (Monks Ärzte im Netz GmbH) will regularly send pseudonymized datasets to the evaluator. These data will be entered into a database. The recruitment numbers will be closely monitored. To visualize the monitoring of recruitment numbers, recruitment graphics will be generated, indicating the recruitment status compared to the target state. Additionally, the data will undergo regular plausibility checks to proactively address any potential incompleteness, double data entries, implausibility, or systematic errors. This can involve adjustments to the user interface, informational materials, or training for the involved gynaecologists and paediatricians. The results of the recruitment monitoring and plausibility checks will be reported to the consortium leadership in a report format.

### Confidentiality {27}

The project is currently hosted on servers that are accessible directly from the internet. The servers are operated in an ISO 27001-certified data centre in Germany and are accessible only to selected personnel of the telemedical service provider. Patient data is encrypted using current encryption methods in accordance with the technical guideline TR-02102–1 of the Federal Office for Information Security (BSI), ensuring that only doctors and patients can access this information. The medical data collected from the questionnaires will be pseudonymized and forwarded to the evaluators only after explicit consent from the patients.

### Plans for collection, laboratory evaluation, and storage of biological specimens for genetic or molecular analysis in this trial/future use {33}

In UPlusE, no biological samples will be taken, and no laboratory tests will be conducted. Only data through questionnaires will be collected.

## Statistical methods

### Statistical methods for primary and secondary outcomes {20a}

The intervention in UPlusE will be scientifically evaluated using a multidimensional approach and various methods (multilevel analyses, process analyses, secondary data analysis) that, in combination, allow for a comprehensive assessment of different aspects of the project.

All data analyses will be conducted using pseudonymized data. After the observation period (U6), the primary and secondary endpoints for parents with psychological and psychosocial issues will be analysed in a group comparison (comparison between the intervention and the control group). Multilevel analyses will be conducted based on the intention-to-treat principle.

Additionally, the trajectory of values and the utilization of services for parents without psychological and psychosocial issues at the time of project inclusion (clinical evaluation) will be descriptively analysed. Quantitative parameters will be analysed using multilevel analyses to correct for cluster randomization. In the case of structural inequality between the intervention and control groups, relevant parameters (such as age, gender, number of pregnancies and births, partnership, socioeconomics—education, occupation, financial situation, severity of depression) will be adjusted for. All tests will be two-tailed with a significance level of alpha = 0.05.

In the analysis, a descriptive evaluation (e.g. symptom severity, child symptoms, service utilization) will be conducted for the group of parents who were positively screened. At the time of U6, a comparison between the intervention and control groups will be performed (e.g. symptom severity, parent–child bonding, child symptoms). Furthermore, across groups, psychosocial stress, depressive symptoms, stigma, and service utilization will be assessed.

The data from the process analysis questionnaires will be analysed descriptively and inferentially.

In addition to the quantitative analysis of utilization, gathered through self-report questionnaires in the app, utilization of healthcare services within the healthcare system and the associated costs triggered by utilization will be descriptively analysed based on billing data from participating health insurance company. Initially, the utilized services will be examined, followed by an examination of the costs of the services used. For the participating health insurance company, the costs of provided services are billed by healthcare providers. These costs are visible in the billing data, which can differentiate between total costs, costs for outpatient and inpatient services, and medication costs. All three participant groups (parents with psychological and/or psychosocial issues in the intervention and control group, as well as parents without psychological and/or psychosocial issues) will be compared in this regard. Both routine data for psychiatric/psychotherapeutic and somatic conditions of parents and their children will be analysed (inpatient and outpatient treatments, emergency responses, medications).

### Interim analyses {21b}

After the recruitment phase is completed, baseline assessments will be performed. Particularly relevant here is the analysis of structural equivalence among participants from the intervention and control practices.

### Methods for additional analyses (e.g. subgroup analyses) {20b}

As sensitivity analyses, per-protocol analyses will also be conducted. Per-protocol analyses consider only the results of those patients who received the intervention according to the study protocol. In the UPlusE project, per-protocol analysis will be performed with data from patients who were positively screened and actually sought assistance.

### Methods in analysis to handle protocol non-adherence and any statistical methods to handle missing data {20c}

As this is a study in a healthcare setting and not a clinical trial testing a clinical procedure or medication, protocol non-adherence is not relevant here. For intention-to-treat analyses, it may become necessary to impute missing data because the intention-to-treat analysis includes all participants, regardless of their adherence to the study. If (potentially multiple) imputation of missing data will be required, it will be carried out especially in the analysis of the primary research question. Scores will be imputed, not individual items, and this will be done separately for each outcome or research question. For per-protocol analyses, missing data will not be a concern, as only participants who have completed all necessary measurements will be considered here. If significant group differences will occur at baseline, the inverse probability weighting procedure may become necessary.

### Plans to give access to the full protocol, participant-leveldata, and statistical code {31c}

Access to pseudonymized data and statistical codes can only be granted within the scope of participants’ consent declarations upon a reasonable request.

## Oversight and monitoring

### Composition of the coordinating centre and trial steering committee {5d}

The UPlusE project will be implemented by a consortium (UPlusE consortium) consisting of members Nuremberg Clinic and Paracelsus Medical University (PMU), Department of Psychiatry, Psychotherapy, and Psychosomatics at the University Hospital Würzburg, Institute for Community Medicine at the University Medicine Greifswald, Chair of Social Paediatrics at the Technical University of Munich, the BKK Landesverband Bayern (including the BKK Vertragsarbeitsgemeinschaft Bayern (VAG)); the BVKJ-Service GmbH; the Sanakey Group; and the ÄVGD Ärztliche Vertragsgemeinschaft Deutschland GmbH.

The principal investigator and study leadership is overseen by Dr. Susanne Simen and Prof. Christoph Fusch of the Nuremberg Clinic and Paracelsus Medical University (PMU).

Consortium partners responsible for implementing the intervention include the Department of Psychiatry, Psychotherapy, and Psychosomatics at the University Hospital Würzburg; the BKK Landesverband Bayern (including the BKK VAG); the BVKJ-Service GmbH; the Sanakey Group; and the ÄVGD Ärztliche Vertragsgemeinschaft Deutschland GmbH.

The Nuremberg Hospital and the University Hospital Würzburg are responsible for CME-certified further education for participating gynaecologists, paediatricians, and mental health practitioners. Both locations actively promote UPlusE to gynaecologists, paediatricians, as well as mental health practitioners and early intervention professionals to garner acceptance and participation.

The primary responsibilities for recruiting gynaecologists and paediatricians for UPlusE lie with the ÄVGD Ärztliche Vertragsgemeinschaft Deutschland GmbH and the BVKJ-Service GmbH. They also manage the billing of UPlusE services with the participating doctors.

The BKK provides and advertises for UPlusE among its insured persons.

Consortium partners for the evaluation include the Institute for Community Medicine at the University Medicine Greifswald and the Chair of Social Paediatrics at the Technical University of Munich. The Institute for Community Medicine also takes on the responsibilities of data management of the pseudonymised data and monitoring of recruitment numbers and collected data. Reactions to that can involve adjustments to the user interface, informational materials, or training for the involved gynaecologists and paediatricians. Many stakeholders and Public Involvement Groups are involved in UPlusE as cooperation partners: Prof. Sarah Kittel-Schneider, Department of Psychiatry, Cork, Ireland; BVKJ; BVF; DGPFG; Marcé-Gesellschaft; DGPPN; BVDP; BVDN; BDK; ackpa; LIPPs, e.V.; NCAD; Deutsche Depressionshilfe; BptK; NZFH; BV pro familia; BKE; Gesundheits- sowie Familien-Ministerium Bayern; Schatten & Licht e.V.. The latter is a lived experience organization focused on peripartum mental health disorders. UPlusE also has an advisory board, which includes representatives from midwifery science, the Department of General Practice at the University Hospital Würzburg, and the Prevention in Childhood and Adolescence Working Group at UKE Hamburg. Their role is to consult on specific topics and to provide insight into stakeholders’ opinions and experiences. They also help to advertise UPlusE among their members and peers.

### Composition of the data monitoring committee, its role, and reporting structure {21a}

UPlusE inheres a very low health risk for study participants. For this reason, a data monitoring committee (DMC) is not needed.

### Adverse event reporting and harms {22}

As mentioned further above, UPlusE is an intervention with a non-invasive nature and risk of stigma due to this screening appears to be very low. Hence, damages or harms are not anticipated. Nonetheless, it could be possible that some participants feel stigmatized harassed or pressured by the intervention. To detect possible adverse events, participants will be asked for feeling stigmatized by questionnaires at enrolment and close out at U6 (process evaluation). Throughout the study, all adverse events and unintended effects will be assessed and documented and countermeasures initiated if necessary.

### Frequency and plans for auditing trial conduct {23}

The results of the recruitment monitoring and plausibility checks will be reported to the consortium leadership in a report format. Monthly consortium meetings will be held, during which recruitment numbers will be discussed as a fixed agenda item. If recruitment is seriously lacking behind, then the project will be opened to insured individuals from all health insurers through treatment contracts according to § 630a of the German Civil Code (BGB). Furthermore, the authors have no competing interests. All study results will be published.

Independent evaluation of the study will be conducted by the Institute of Community Medicine and the Chair of Social Paediatrics at the Technical University of Munich, which both have an external and independent role to the principal investigator and sponsor.

### Plans for communicating important protocol amendments to relevant parties (e.g. trial participants, ethical committees) {25}

We will communicate protocol modifications and relevant process changes to the Local Ethical Committee at the University Medicine Greifswald and to all relevant ethical committees of the study partners. All changes will be noted in the study registration as well.

## Dissemination plans {31a}

Every year during the project period, status seminars will be held, where investigators, participants, healthcare professionals, the public, and other relevant groups will be invited and where (interims) trial results will be presented. Results will further be published in scientific journals as open-access publications and will be stated in the study registration.

## Discussion

Depression and other mental health burdens as well as psychosocial distress and impaired bonding are often unnoticed during the peri- and postpartum period [5, 18]. Gynaecologists and paediatricians regularly encounter individuals in this phase of life. Even though the guidelines for children and maternity care consider the need to recognize psychosocial burdens, physicians frequently fail to recognize the psychological burdens of the parents [18]. Paediatricians are specialists for children. The parents of the children are, strictly speaking, not their patients. Both, gynaecologists and paediatricians, are not trained deeply in diagnosing and treating mental health conditions and therefore have mostly insufficient knowledge, competence, time slots, and the tools to recognize and to respond to depression and psychosocial burdens in mothers/parents. UPlusE will provide the necessary knowledge through CME-certified online training and offer the instrument for a systematic and standardized screening of depression and psychosocial burdens. Gynaecologists and paediatricians will then be able to comply with the guidelines for children and maternity care. Because UPlusE will be using the already widely spread practice apps ‘My Gynaecology Practice’ and ‘My Paediatric Practice’, we anticipate a high willingness to participate among physicians and a relatively low additional workload in everyday practice.

In a regional pilot project in Nuremberg, over the course of 21 months (from April 2020 to December 2021) during the SARS-CoV-2-pandemic and without additional compensation, *N* = 5235 women were screened in gynaecology and paediatric practices using the EPDS [25]. The acceptance among both gynaecologists and paediatricians was very high, feasibility found to be good. The EPDS also included questions about traumatic birth and experiences of violence. Overall, a prevalence of pregnancy depression (defined using an EPDS cutoff ≥ 10) was found to be 9.94%, with 43.9% of women having an EPDS total score > 12. The prevalence of postpartum depression was 10.18%, with 46.9% having an EPDS total score > 12; 10.87% of women reported experiences of violence, while 19.64% of postpartum surveyed women reported a traumatically experienced birth; in contrast, 4.53% indicated both experiences of violence and a traumatically experienced birth. The following statements about the pilot project were documented by the participating paediatric and gynaecology practices: ‘I was surprised by the percentage of mothers showing symptoms – thinking back to the time before screening in terms of undetected mothers,’ ‘The patients are very satisfied with the screening – they appreciate being asked about their mental well-being,’ ‘What's remarkable is that a significant number of affected mothers only discuss it openly after the questionnaire is addressed due to conspicuous responses,’ ‘The plus-questions help identify pregnant women who require special trauma-sensitive care during childbirth’. Mothers found it indeed helpful to be addressed about a conspicuous result in the questionnaire. Overall, the dropout rate was very low (1.22%). In a survey conducted by Schatten & Licht e.V. (self-help organization for peripartum mental disorders), 97% of the affected individuals (*N* = 58) endorsed the screening as described above. As a conclusion for UPlusE, a high acceptance among healthcare providers and patients can be inferred [25, 29].

With utilization as the primary outcome, UPlusE will provide evidence that more mothers/parents with depression and/or psychosocial burdens seek treatment or counselling. We also expect to provide evidence for an improvement of mental health in young families, an increase of unimpaired parent–child relationships, a decrease of psychosocial burdens, and an improvement of regulatory problems in children.

A potential selection bias cannot be excluded, since data are collected from app users only. These are mostly German speaking patients since the app and the study information are only available in German language.

What should further be considered a limitation of the study is that only one partner (mother or father) can be registered and fill out questionnaires in the Practice App. Accordingly, routine data from the participating health insurance can only be provided for this partner and only if they are insured with this health insurance. Long-term observation beyond the first year of the children’s lives is currently not possible. However, this would be highly desirable, as parental depression, in particular, can have lifelong effects on the development of children. The further development of healthcare costs and other non-health outcomes, such as academic progress, would also be very interesting to explore over time.

## Trial status

This is the first and original version of the UPlusE study protocol (version from 15 July 2024). The recruitment of participants started on 9 February 2024 and will be concluded when 10,000 patients have been recruited or on 30 April 2026.

## Data Availability

Access to pseudonymized data and statistical codes can only be granted within the scope of participants' consent declarations upon a reasonable request and, if applicable, only after reassessment by the ethics committee.

## Abbreviations

ÄVGD: Ärztliche Vertragsgemeinschaft Deutschland GmbH – Medical Contractual Association Germany GmbH
BGB: German Civil Code
BSI: Federal Office for Information Security
BVKJ-Service GmbH: Service GmbH des Berufsverbandes der Kinder-und Jugendärzte e. V. – Service GmbH of the Professional Association of Paediatricians and Youth Doctors e. V.
CME: Continuing medical education
DC: Diagnostic criteria
e.g.: For example
EPDS: Edinburgh Postnatal Depression Scale
G-BA: Gemeinsamer Bundesausschuss – German Federal Joint Committee
ICC: Intra-Cluster-Correlation
KoKi: Koordinierende Kinderschutzstellen – Coordinating Child Protection Centers
PBQ: Postpartum bonding questionnaire
REDCap: Research Electronic Data Capture for creating and managing online surveys and data collection forms
SFS: Questionnaire on Crying, Feeding, and Sleeping
TAU: Treatment as usual
U3: Regular care checkup of the child at the age of 4–5 weeks
U4: Regular care checkup of the child at the age of 3–4 months
U5: Regular care checkup of the child at the age of 6–7 months
U6: Regular care checkup of the child at the age of 10–12 months
UPlusE: U-Untersuchung für Kinder PLUS Eltern beim Pädiater zur Förderung der kindlichen Entwicklung mit Impuls aus frauenärztlicher Schwangerenvorsorge“ – Pediatric Check-up for Children PLUS Parents at the Pediatrician to Promote Child Development with Input from Gynecological Prenatal Care
